# The operational experience of private owners of small-sized care homes in China: a qualitative study

**DOI:** 10.1186/s12913-023-10066-w

**Published:** 2023-10-06

**Authors:** Zhang Xiuxiang, Xia Qinghua, Jennifer K. Quint, Ann D. Morgan, Brendan McCormack, Xiubin Zhang

**Affiliations:** 1 Economics and Management School, Wuhan University, Huanghuai University, 299 Bayi Rd, Wuhan, China; 2 Economics and Management School, Wuhan University, 299 Bayi Rd, Wuhan, China; 3 School of Public Health, Imperial College London, London, UK; 4 Imperial College London, School of Public Health, National Heart and Lung Institute, London, UK; 5 Faculty of Medicine and Health, Sydney University, Sydney, Australia; 6 School of Public Health and National Heart and Lung Institute, Imperial College London, White City, London, W12 7RQ UK

**Keywords:** Private owner, Care home, Interpretative phenomenological analysis, Qualitative research, China

## Abstract

**Background:**

Private small-sized care homes (<50 beds)  have proliferated across China, however, until recently little was known about the characteristics of such institutions, and the challenges and the problems faced by their owners. This study aimed to explore the characteristics of small-sized, privately-owned care homes in the People’s Republic of China; and to understand the motivation and challenges faced by their owners.

**Methods:**

This study used an interpretative phenomenological analysis approach of qualitative research. Owners of eight small-sized private care homes located in two cities of Henan Province, China, were interviewed using semi-structured interviews.

**Results:**

Four themes and eight subthemes were identified: 1. Motivation for establishing a care home business; 2. Certification and establishing a legal footing for the business; 3. Operational challenges; 4. Future business development. The study found that the development of privately owned small-sized care homes faced great challenges and critical survival problems due to policies, staffing, and management issues. There is a lack of regulations about the safety and quality of care provided for older people and a lack of legal protections for the owners of small-sized private care homes.

**Conclusion:**

The study suggests that formal regulations and provisions are needed to support these smaller-sized private care homes. Monitoring is also needed to ensure the quality of care. It also suggests that there needs more support by policymakers as well as provision monitoring services to improve quality of care in these care homes. Care regulations and standards are not unique to China so findings from this study can be applied to places where there are similar situations or if there are aged care services still developing.

**Supplementary Information:**

The online version contains supplementary material available at 10.1186/s12913-023-10066-w.

## Background

In China, the rapidly ageing population and the burden this places on the healthcare system is of increasing concern [1, 2]. Latest estimates suggest that in 2019 there were 164.5 million Chinese citizens aged 65 and over, 26 million of whom were aged 80 and over [3]. It is projected that the proportion of the population aged 65 and over will increase rapidly in the next 30 years, from 11.7% to 24.7% in 2044 [3], reaching a total of 365 million by 2050 [1]. Traditionally, care of the older adults has been viewed as part of a Chinese family’s obligation and duty; younger members of family are expected to provide support and care for their older parents and relatives. However, the combination of rapid socioeconomic change, an ageing population, the one-child policy and other demographic shifts, is eroding the ability and capacity of the family-based system to care for the older population in China [4]. Moreover, long-term care costs for older people are not currently covered by national healthcare insurance schemes [5] and only older people without children are eligible for places in government-funded public care homes [6]. Although some aged care insurance pilot programmes have been implemented in several cities from 2016, studies have since found that such programmes are often fragmented in their coverage, eligibility, funding and reimbursement [4, 5].

Recognising the need for greater provision of long-term care for its ageing population, the Chinese government in its 12^th^, 13^th^ and 14^th^ Five-Year Plans for National Economic and Social Development (2011–15, 2016–20, 2021–25), has outlined its intention to develop a three-tiered long-term care system. These consecutive five-year plans set targets for a so-called 90–7-3 structure for long-term care services, with 90% of older people receiving care in their homes, 7% supported by community-based services and 3% receiving institutional care [6]. However, the 5-year plans simply set targets but do not really describe how these targets might be achieved and more importantly how care is to be accessed and funded for individuals, especially community-based services and institutional care [7]. The bottom line is, although there are some insurance schemes being developed (piloted) they are still limited in scope and most people are unable to access any form of state provision— either community services or institutional care. In practice, therefore, the cost of long-term care is currently largely borne by individuals and families, and for most of the working population, remains unaffordable, especially long-term institutional care.

In addition, the national regulatory framework of long-term care is vague in China. The Central Ministry of Civil Affair Bureau leaves provinces to create and carry out policies and guidelines. The provinces then leave it to the cities and individual counties. So, there are no integrated national regulations overseeing long-term care institutions, practice, the local civil authorities are responsible for overseeing regulations to their local care homes [6, 8].

Many families who find themselves unable to provide care for their loved ones in their own homes and/or unable to access the limited state-provided care are increasingly turning to the private sector [6, 9, 10]. This demand has led to the proliferation of private businesses offering care services for older people [11]. While there has been a growth in the number of larger institutions offering long-term care of the older population, these have tended to be located in bigger, more affluent cities [6, 12]. Elsewhere, private sector provision has been dominated by a growth in small-sized, private care homes which tend to cater to the healthcare needs of older people in the local neighbourhood at much lower cost [1]. This has led to heterogeneity in the size, type and quality of long-term care service provision between rural and urban areas, and between regions at different stages of economic development [13]. It is likely therefore that older people in China have widely differing experiences of care services – in terms the quality of the facilities and the level of medical care provided – depending on where they live and whether they are cared for in large, long-term care institutions or small-sized care homes.

Although private care homes have proliferated across China [8, 14], until recently little was known about the characteristics of such institutions and the challenges and problems faced by their owners. This is especially true of the smaller private care homes which, as previously mentioned, tend to be more common in towns and rural communities. Considering their role in influencing the safety and quality of long-term care services, it is important to better understand the experiences of this currently under-studied population of care home owners. This enhanced understanding would help to improve the regulation, monitoring and level of state support provided to this type of care home, and in turn improve the quality of care provided to their residents. The aim of this study was therefore to describe the characteristics of privately-owned small-sized care homes in China and the operational experience of their owners. The study also aimed to investigate the challenges and issues faced by care home owners in order to inform relevant stakeholders, such as healthcare policy-makers, care home owners and researchers working in the field of care for older adults.

In this study, we defined a small-sized care home as one that had fewer than 50 beds taking ‘Urban Older Adult Facilities Planning Specification’ in China as a reference [15]. Small-sized care homes are less likely to have been officially registered due to the inconsistent registration regulations of the central and local authority, care home size, or for some practical reasons [16]. Typically, these small-sized private care homes are not staffed by qualified healthcare professionals and are more likely to have untrained carers who are only able to provide basic physical care [17, 18].

## Theory

An interpretative phenomenological analysis (IPA) approach was adopted for this study. IPA is a qualitative research method which by focusing on specific events or important phenomena offers insight into how individuals interpret a given phenomenon in a given context [19]. Usually, these phenomena are related to personal experiences, such as major life events or the development of important relationships. IPA has its theoretical origins in phenomenology and hermeneutics, combining psychology, interpretation and specific context [20]. As a research methodology, IPA lends itself to the study of the experience of care home owners as opening a care home is an important life event for those who choose to pursue this option. Furthermore, through careful analysis of participants’ responses, IPA can provide detailed situation-based insights into participants’ experiences and how they made sense of them [21].

## Methods

### Participant recruitment

The research was undertaken in Henan Province, China. Compared with other Chinese provinces, Henan is culturally rich but less economically developed. It has a population of more than 100 million, of which 11.2% are aged 65 years or more [22]. To be included in the study participants had to be aged 18 years or over and own a small care home (less than 50 beds). A snow-balling approach was used to recruit participants. The first participant was recruited through a neighbourhood social event by the first author (whose research expertise is in field of small business management and who has close links with entrepreneurs with interests in long-term care service provision); the remainder were introduced one by one. Eight care home owners from two cities in Henan agreed to participate in the study.

### Data collection

Semi-structured interviews were conducted by the first author, who is an experienced mixed-methods researcher. Open-ended questions were used in allow participants’ free expression of their experiences and viewpoints [23]. For example, to explore how care home owners responded to a particular situation, we asked questions such as: “How has the experience of enterprise changed your life?” and “what the meaning of running a care home to you?” Likewise, questions aimed at understanding what challenges owners faced when starting their business, we used questions such as: What challenges did you face?” (See appendix 1: Interview Guide).

The face-to-face interviews lasted for 1.0–1.5 h and were audio recorded for later transcription. A digital recorder was used to record the conversations. Field notes were made; these focused on recording participants’ non-verbal signs and important reactions and were used as supplementary materials to the interview datasets. Data saturation was reached with the eighth interview as no new themes emerged; data collection was thus stopped at eight interviews.

### Data analysis

Two researchers (ZX, XQ) conducted the data analysis which followed the six-step IPA process outlined by Smith and Osborn [24]. Firstly, both authors read and reread the transcripts to gain familiarity with the participants’ stories. Secondly, an initial set of codes (reflect the source material) that emerged from a line-by-line analysis of the texts. Thirdly, the preliminary codes were sorted into potential themes by grouping them together according to conceptual similarities, and all relevant coded data extracts grouped within the identified initial themes. At this stage, the two researchers created the codes independently, then reliability checks were conducted by matching the initial codes with their coded extracts. Fourthly, in line with standard IPA methodology, the data sets were analysed one-by-one. Once all the transcriptions had been analysed to identify the patterns and connections between the datasets, the initial set of themes were then developed into a thematic map (step 5). Finally, a consistency check was conducted by two research members independently; any inconsistencies were discussed within the wider research team and agreement reached, after which the final list of super-ordinate themes and subthemes was drawn up by two researchers working together, and compelling extracts selected. The findings were returned to the participants for feedback and confirmation. NVivo11 software was used for data management and data analysis.

## Results

### Characteristics of the social entrepreneurs and the care homes

Study participants ranged in age from 38 to 65 years. Two care home owners were educated to university level, four had completed secondary school, one had only attended primary school and one had received no formal schooling. Participants had spent between 1 and 13 years running their care homes. The smallest care home had just 10 beds, while the largest was able to accommodate up to 50 residents. Most were run as a family business, providing basic physical care in a “home-like” environment. In the words of one participant:

“My nursing home is just like home. We have no standardized rules, while our nursing home is more casual, the old people are just like at home. This way they are also comfortable.” (P1).

Only two care homes were formally registered and certified by the local Civil Affairs Bureau. Although the other six had not been issued with a certificate or other form of licence to operate as a care home, they had been given informal permission by the local Civil Affairs Bureau. The cost of care provided by the care homes included in this study was lower than that charged by larger, and fully licensed, health care institutions.

All the care homes are private and overseen by the local civil authorities. The older people who live there pay for the services (care cost) by themselves or their families pay it for them.

Four key themes and eight subthemes emerged from the analysis of the data generated by the semi-structured interviews, as follows: 1. Motivation for establishing a care home business (looking after a family member while earning an income, job redundancy, following the example of others); 2. Certification and establishing a legal footing for the business (local authority registration, impact of local planning policies); 3. Operational challenges (staff recruitment, low profits, lack of standardized regulation and operational guidelines); 4. Future business development.

#### Theme One: Motivation for establishing a care home business

Participants articulated a number of reasons for opening a care home.

##### Looking after a family member while earning an income

In this study, four participants opened the care homes because they needed to care for their parents or other older family members. In this respect, participant 2’s response was typical:

*“I want to open a care home because there are three or four old family members that needed to be looked after, my mother, my mother-in-law and my father-in-law. Other relatives and friends also have somebody that needed to be looked after. So, they all said, ‘why don’t you open a care home’? Therefore, to support our family, to take care of the old as well as to take care of our children, I started this care home.” (P2)*

In China, cultural norms dictate that children take care of their dependent older parents in the family home. Caring for an older parent means providing both physical care and economic support, and often necessitates one family member committing to staying at home to look after their loved one(s). Some may have to relinquish paid work outside the home in order to fulfil their filial duty, leaving a shortfall in the family income. The need to find a way to bring in extra income was cited by four participants as being the main reason why they started a home-based business. For example, one said:

*"I started the home because my mother had dementia. I couldn't afford to send her to a care home. So, I thought, why can’t I start a care home by myself? I can look after my mother at home while bringing income for my own family." (P3)*

Several study participants highlighted the twin objectives of seeking affordable care for their older parent while simultaneously generating income for the whole family as a key motivator behind their decision to open a care home. As one participant explained:

*“13 years ago, my husband had a cerebral haemorrhage and I couldn’t go out to work. My children were still young... I had to start this care home. I mean, you cannot look after your family when you go to work, can you? It was 13 years ago, it was 2007.” (P1)*

In P1’s case, operating her care home within her family house allowed her to look after her children while running a business at the same time.

##### Job redundancy

Redundancy was mentioned a reason for opening a small-sized care home by two participants. In China, if redundancy happens in middle-age, re-employment is often difficult to achieve. According to one interviewee, being made redundant from the army was the primary reason behind his decision to develop his private care home business:

*“I was young at the time, only 40 years old when I came out of the army. It was impossible to leave the cadre without doing anything, and anyone would be mad just staying at home, yes, right? I thought, I should do something, so I started it.” (P5)*

##### Following the example of others

Several participants said their decision to start a care home was prompted by the experience of family members or friends who had set up similar businesses. For example, participant 6 said:

*“In my husband’s family, my mother-in-law runs a care home; my sister-in-law and my uncle-in-law each run a care home. In the beginning, I helped my mother-in-law in her care home, I have learned a lot. I thought about opening a care home by myself as I would earn more money than working as a carer. From last year, I started a new care home by myself.” (P6)*

Participant 4 recounted how his care-home enterprise arose from a causal conversation with colleagues:

“There was a boss who had set up a nursing home. Once, I had dinner with my colleagues, one of my colleagues in the army said, ‘Ah, there is a boss who runs a nursing home, are you interested?” I didn’t know everything at that time, but I was interested in having my own business, so I decided to try it.” (P4)

In sum, in this study most participants cited either personal reasons (the need to look after older family members or friends) and/or economic necessity as the key factor behind their decision to open a care home. For several, operating care home served a dual purpose (providing an opportunity to look after an older family member while also earning an income); for others, the decision to establish a care home business was purely economic, borne out of a desire to start their own business, for example, following redundancy or following the example of others.

#### Theme two: Certification and establishing a legal footing for the business

##### Local authority registration

Despite expressing a desire to do so, many study participants cited difficulties in registering their business with the local authorities. Most of the licensing difficulties were directly related to fire safety certification. In China, it is a legal requirement for a care home to obtain a fire certificate. However, most owners of small-sized care homes, including those who participated in this study, either use their own houses for their businesses or rent buildings from private landlords. It is unlikely therefore that their premises would have had fire equipment installed, especially given that buildings rented to care home owners are often unfit for other business purposes. Moreover, merely installing a fire system is usually not enough to meet the requirements stipulated by the local fire bureau. Owners thus often face multiple barriers to obtaining a fire certificate which would allow them to run their care homes legally. Because of the high demand for small-sized private care homes which offer local residents affordable and convenient care for their loved ones, what happens in practice is that the local civil bureau allows care homes to open but does not authorise their registration.

Participant 2 explained the situation that they faced as follows:

*“I feel stressed about the fire certificate, we are not like big care homes, they can get a fire certificate. If the fire bureau gives us a fire qualification certificate, the nursing home will be legalized and formalized, it will be better.” (P2)*

Other participants expressed similar concerns and issues in obtaining a fire certificate:

*“The fire certificate here is required by the local Civil Affairs Bureau, but the local Fire Department does not install fire facilities for us, the fire department did not implement a central policy, or they have not implemented it at the moment. At the very least, they would talk about various issues and find an excuse.... the fire certificate is difficult to gain.” (P3)*

Participant 6 described a similar problem: even though he was encouraged to use his own property as a care home, his building was deemed not suitable for business usage. When talking about this, he seemed a bit confused and said:

*“There are two problems, …the second one, is that this is a self-built house. The local fire department cannot issue a fire certificate to me, there is no way to get a certificate registration. Well, when I built the house, the local government encouraged me to open a care home in a private house. But it seems that this kind of thing conflicts between these two departments.” (P6)*

##### Effects of local planning policies

Local planning policy was identified as a second area of tension between care home owners and local authorities. Local government plans to demolish old buildings in order to improve infrastructure has meant that owners of care homes were finding it increasingly difficult to find suitable buildings and locations where they could develop their businesses. For example, one participant said:

*“The government said it will be demolished, this area will be demolished, I have no choice, it is unstable.” (P7)*

Participant 5 also voiced concerns about the potential threat of demolition:

*“I am afraid of demolition. I will not be able to open it. Now houses are hard to find. Our homeland will soon be gone. I originally wanted to build a house. There is no place to build a house for the care home. I don't even know what to do if it will be demolished here.” (P5)*

These extracts highlight the potential impact of a second area of tension between care home owners and local authorities which arises out of local government ambitions to re-develop run-down areas. Faced with the threat of demolition, many participants voiced concerns about the future of their care home businesses.

#### Theme three: Operational challenges

All study participants spoke of the multiple challenges they faced in running a care home.

##### Staff recruitment

Among the challenges most frequently cited were problems with staff recruitment, staff management and business profitability. None of the eight care homes included in this study were staffed by qualitied nursing staff. The caregivers were mostly uneducated. As Participant 5 explained the lack of appropriately trained staff represented greatest challenge to the safe operation of their care home:

*“I think the biggest problem is the care staff problems. We have no qualified staff. You know, the older people are more vulnerable. The care staff can't respond well if there are some unexpected situations that happen with the older person.” (P5)*

Participant 3 voiced similar concerns, which she believed stemmed from an inability to recruit younger staff (due to the low pay and the poor reputation of the care sector) forcing her to rely on older, uneducated staff:

*“The care staff are all in their 50s or 60s, and even if we give them training, their acceptance to learn new knowledge is relatively low. They were reluctant to study and some of them were illiterate, they did not understand. So I think care staff is the biggest problem.” (P3)*

Participant 2 had a different experience with staffing problems. She recruited two caregivers who were from rural areas. She described the difficulties she faced during harvest times as follows:

*“Some of the carers are from rural villages, and so they have to go back home during the autumn harvest season and in the early summer for harvesting wheat. During these periods, they have to go back. When they leave, I must find someone again. Sometimes I can't find the appropriate person.” (P2)*

She went on to say that on one occasion she and her husband had to look after the residents by themselves when the staff left for harvest time because she could not find anyone to step in. Participant 4 also talked about the issue of staff; he said:

*“This job is not easy to do, because the care staff are not easy to find. I was left for a month without care staff. It is hard to recruit care staff as the salary is low. We cannot afford it if the salary is too high. I am looking for care staff over 55, about 60 years old, according to their age, they are more likely to be retired, because their family is not in a good condition, so they come out and do this job to bring more money in. I am also looking for people from the rural areas.” (P4)*

These extracts not only illustrate the range of difficulties in recruiting trained staff, but also how common staff recruitment problems are in this sector. Lack of availability of well-qualified staff may also be contributing to challenges associated with poor recruitment, low profitability and poor quality of care in private, small-sized care homes.

##### Low profitability

All participants, without exception, commented on the low profitability of their care home businesses. For example:

*“Oh, not much. Not much profit. Our charges are too low. I have to pay the carers and the chef, buy food, and pay the fees of water and electricity and everything else. The profit is only 2,000 yuan per month.” (P5)*

With profits on a par with the average salary of a migrant worker (about 2000–3000 yuan), it is not surprising that participant 5 further commented that financial gain was not her main motivation for opening her care home but rather the ability to look after her own family while simultaneously running her business.

##### Lack of regulations and guidelines governing standard of care in care homes

As there lack standardized regulation, there is more likely presented some operational conflicts in these care homes. For example, participant 2 said:

*“… If something happens in a nursing home, it is similar to a war, especially when it comes to older people in critical conditions. I do not dare to go far, do not dare to travel, do not dare to leave too long.” (P8)*

In this extract, because this care home is not bound by regulations or guidelines which outline what care standards should be followed, they can be sued by older people if something happens to a resident for whom they care. As a result, the owner must stay in the care home on a continuous basis to monitor everything. This not only reflects arrangement in a crisis, but it also highlights staff management problems.

During the course of her interview, participant 2 disclosed a suicide event, which had caused a considerable amount of distress and which led her to consider closing her business. She described the situation and its consequences as follows:

*‘I had to pay five hundred thousand yuan to the family (the older person committed suicide in the care home) even though it is not my fault, I was scared and disappointed, I was determined to close down the care home.” (P2)*

In this case, the matter was settled between the care home and the deceased person’s family through financial payment with no formal investigation by the appropriate authorities. This event serves to highlight the disadvantages of a system based on unregistered care home provision and the need for regulations that protect both the residents of care homes and the care home owners.

#### Theme four: Future business development

In view of the many issues and difficulties surrounding the running of their care home they had experienced, five of the eight participants indicated that they were planning to close their care home. Participant 2 cited the imminent threat of demolition as the reason for her decision to close her care home; she said:

*“We rented this site. We stayed here for more than five years. We could not predict that the Qingshan Scenic Area would develop into a five-star scenic spot and that we can’t stay here anymore. Oh, I am no longer looking for a new site.” (P2)*

Participant 5 said his decision to close his care home stemmed from problems meeting fire department rules. Another participant reported an issue regarding finding a suitable site for his care home:

*“Yes, if you don't have a suitable site, the rent can be very high. You see, now, why there are no nursing homes around, because no one rents the house to you, even if you have money, people do not rent it to you. They think that why are you willing to take care of the old people as it isn’t a profit business.” (P7)*

Participant responses reflect a range of concerns relating to the viability of running successful care home, including difficulties in finding suitable premises and low profit margins. Low profitability represented the greatest threat to the sustainability and future prospects of participants’ care home businesses.

Only one owner described plans to expand her care home business:

*“I plan to open another branch. I would like it to be bigger, and I don’t want it to be small anymore, because the management of a small staff team is also more troublesome. I would like it to have a greater number of beds with qualified care staff.” (P6)*

In this study, most of the participants were pessimistic about their ability to develop their care home business; this poses a considerable challenge for policy-makers seeking to grow this sector to meet increasing demand for long-term for the older adults.

## Discussion

This study sought to understand the experiences of owners of private, small-sized care homes in Henan Province, China. It explored owners’ motivations for opening a care home and the challenges they faced running their care home business. This study revealed that financial gain was not a primary motivation for opening a care home; most participants opened their care homes out of necessity, the major reason being a need to take care of older family members or people in the neighbourhood. Starting a small-sized business was often perceived as a way to combine earning an income with looking after the family.

In terms of challenges, several owners mentioned financial issues as being their main concern. Participants described having to rely on their own funds or savings, family resources or loans from relatives and friends to provide the starting capital to open their care homes. More than half of participants said that the profit they made was less than the local average salary; three said that low profitability was a major problem for them, threatening business sustainability and making it difficult for them to keep their business going. These findings suggest that there is an urgent need for relevant stakeholders, such as government policy-makers and local authorities, to consider how they can best support private, small-sized care homes which provide much needed long-term care for older residents who, for whatever reason, cannot be cared for by their families at home.

The inability to recruit appropriately trained staff was another key challenge identified by the owners of small care homes who took part in this study; several participants believed that this compromised the quality of care they were able to provide. None of the eight care homes included in this study employed qualified healthcare professionals; a high proportion of care staff were rural migratory workers with a low level of educational attainment. None had received any pre-employment training. Other studies conducted in China have also found that the majority of private caregivers were unskilled and uncertified, and had received little or no training from their recruitment agencies [6, 25]. The literature also indicates that problems associated with staff recruitment and quality of care in long-term care settings are not unique to China but exist across the globe [26–29]. While our study is too small to allow transferable conclusions to be made, it serves to highlight the current situation as it relates to staffing issues and quality of care provided in private, small-sized care homes in China. Moreover, it underscores the need for policy-makers and care home managers to make this a priority by taking steps to increase the size of an adequately trained care workforce and to implement care quality monitoring in order to improve the quality of care provided.

Our study has also highlighted the lack of coherent, integrated government policy surrounding long-term care provision at a local level. Previous studies have shown that legal regulations are more likely to be neglected in the private care sector than the public care sector in China [30, 31]. During the period of economic development and social transformation in China, many policies and programmes have been implemented independently and are administered by separate local authorities [1, 31]. Consequently, and as reported in previous studies, standard operating policies and regulations governing private care homes do not currently exist [8, 30, 32, 33]. As this study demonstrated, this can have serious ramifications for care home owners. Several study participants faced licensing problems due to the lack of cooperation between the local fire department and the Civil Affairs Bureau. For one participant, the absence of regulatory guidelines resulted in a particularly distressing situation when one older resident committed suicide, a situation which was ultimately resolved through a financial settlement. This particular case underscores the need for greater consideration of issues related to the safety and quality of aged care, especially in small-sized, private care homes in China. As populations age, demand for care services for older people is increasing dramatically worldwide. As elsewhere, the Chinese government is addressing and responding to this issue, and has recently completed its Ten-year Health-care Reform project [1]. Many local governments, especially those in larger, more industrialised cities, are prioritising the funding and development of its long-term care capacity. For example, Beijing is raising funds and providing more support for its older residents [34]. However, in the less economically developed and thus less well-resourced areas, it is more difficult for local governments to take responsibility of caring for their senior citizens. One way to tackle this issue is through nationally-administrated standardised regulations, professional guidance and quality surveillance, coupled with funding support and assistance to reduce geographic disparities in provision of long-term aged care services. Another way is to establish a nationally integrated care funding system to support the growth of private aged care services.

As our study population was composed of officially registered (*n* = 2) and unregistered care homes (*n* = 6), their experiences may differ. Unregistered care home owners experience issues such as the threat of demolition and liability, which may be different from registered care home owners, this may be worth exploring further. At the same time, lacking legal registration could mean lacking legal protection for the care home owners too. Our study explored the care home owners’ experiences of opening a small-sized care home, they may perceive different experiences from large care home owners. There is potential to conduct a study to compare the different experiences of these two types of care homes in the future. This study also has a number of other limitations. First, the IPA study design prohibited the recruitment of a larger sample and so the findings may not be transferable to a larger population. Moreover, this study was conducted in two small-sized cities and as such does not reflect the greater heterogeneity of the care home owners in big cities. In addition, the study was conducted in a single province (Henan Province); this too has implications for generalisability of our findings given that the experiences of our study participants may differ from those from other provinces where different local regulations and rules apply. Nevertheless, by allowing participants to express their experiences of running a care home from their perspective, this study serves as a ‘window’ into the challenges and problems faced by owners of private, small-sized care homes.

## Conclusion

This study describes the characteristics of small-sized care homes and the operational experience of their owners in two cities in China. Despite the huge and rapidly expanding demand for private care homes in China, without exception participants’ responses were dominated by voicing multiple challenges and concerns, including problems with staff recruitment, financial insecurity and difficulties in obtaining the necessary licences and certifications to operate a care home legally. Many described having to work extremely hard for small financial gains and the financial and business developmental risks they were having to take simply to run their business. These findings are of concern as these issues, if not resolved, may act as deterrents to the survival and ongoing development of the private care home sector.

Private, small-sized care homes will likely play a greater role in providing care services as the population ages and the socioeconomic climate of contemporary China changes. Based on the findings of this study, there is a need for the government to provide more political and financial support for privately operated care homes, as well as to take more responsibility for legislating and monitoring the quality of care provided in this sector. The lack of profitability also needs to be addressed if the private sector is to help fill the gap in long-term care services for the old adults. The disparity between services supply and demand for care is not one that is confined to China but is an issue that needs to be addressed everywhere as the world’s population continues to age.

### Supplementary Information

**Additional file 1: Appendix 1.** Interview Guide.

## Data Availability

Data are not able to be obtained from a third party and are not publicly available. The full dataset and data analysis code following receipt of ethics approval may be available from the corresponding author XZ.
