# Correction: The operational experience of private owners of small-sized care homes in China: a qualitative study

**DOI:** 10.1186/s12913-023-10239-7

**Published:** 2023-11-15

**Authors:** Zhang Xiuxiang, Xia Qinghua, Jennifer K. Quint, Ann D. Morgan, Brendan McCormack, Xiubin Zhang

**Affiliations:** 1 Economics and Management School, Wuhan University, Huanghuai University, 299 Bayi Rd, Wuhan, China; 2 Economics and Management School, Wuhan University, 299 Bayi Rd, Wuhan, China; 3 School of Public Health, Imperial College London, London, UK; 4 Imperial College London, School of Public Health, National Heart and Lung Institute, London, UK; 5 Faculty of Medicine and Health, Sydney University, Sydney, Australia; 6 School of Public Health and National Heart and Lung Institute, Imperial College London, White City, London, W12 7RQ UK

**Correction to:** ***BMC Health Services Research*** **(2023) 23:1069**

10.1186/s12913-023-10066-w

Following publication of the original article [1], the authors identified an error in the given name of the fifth author.

The incorrect author name is: Branden McCormack.

The correct author name is: Brendan McCormack.

The author group has been updated above and the original article [1] has been corrected.
