# Supplementary material for: The operational experience of private owners of small-sized care homes in China: a qualitative study
Source: BMC Health Serv Res. 2023 Oct 6;23:1069. doi: 10.1186/s12913-023-10066-w (PMC10559394; doi:10.1186/s12913-023-10066-w)
Supplement: Supplementary file 1 — Additional file 1: Appendix 1. Interview Guide. [file 12913_2023_10066_MOESM1_ESM.docx]

**Appendix 1**

**Interview Guide**

**Study Title:** The operational experience of private owners of small-sized care homes in China: a qualitative study

Welcome from the moderator:

Thank you all for being here. Today we would like to hear your story of opening your care homes and to understand your experience of running a care home. I will be leading the group through the discussion and moderating it. My colleague will be taking notes and recording the session. We want to listen to each of you in order to get your views. At times I will ask for more information or ask for your opinion if we have not heard from you. The session will last up to an hour and will be audio recorded.

Do you have any questions before we begin?

**Introductory/Engagement Questions**

Asked right at the beginning of a focus group session, introductory questions should be “something that elicits stories about a common experience,” stimulating the participants to engage one another and quickly foster a sense of community, to get participants talking about the research topic – such queries should be general and easy to answer.

1. When did you start your care home? Please describe your experience.

**Exploration Questions**

This type of focus group question is used to dive deep into the research topic and elicit detailed responses from participants that offer insight into their needs, wants, and concerns. These questions should be structured to draw out as much information from members as possible.

1. Under what circumstances and how did you come up with the idea of starting a care home? (including the time and reasons)

2. What is the meaning of running a care home to you?”

3. What are the motivations for establishing a care home business?

4. What are the challenges in starting a care home business? (the obstacles and resistance?) what happened, what did you do?

5. How has the experience of opening a care home changed your life?”

6. Is the current business venture different from the original plan and goal? Why? What do you think?

7. What kind of support do you think you need at the moment?

**Follow-up Questions**

What do you mean when you say “X”? - Can you briefly describe it? - What did you do when that happened? - What do you think it is about X that makes you feel that way?

**Exit Questions**

Is there anything else you’d like to say about today’s topic? Any additional questions?
